# Influence of PBL with open-book tests on knowledge retention measured with progress tests

**DOI:** 10.1007/s10459-012-9386-8

**Published:** 2012-06-27

**Authors:** M. Heijne-Penninga, J. B. M. Kuks, W. H. A. Hofman, A. M. M. Muijtjens, J. Cohen-Schotanus

**Affiliations:** 1 Institute for Medical Education, University of Groningen and University Medical Center Groningen, A. Deusinglaan 1, 9713 AV Groningen, The Netherlands; 2 University Centre for Learning and Teaching, University of Groningen, Landleven 1, 9747 AD Groningen, The Netherlands; 3 Faculty of Health, Medicine, and Life Sciences, Maastricht University, PO Box 616, 6200 MD Maastricht, The Netherlands; 4 Centre for Research and Innovation in Medical Education, University of Groningen and University Medical Center Groningen, Groningen, The Netherlands

**Keywords:** Assessment, Knowledge, Open-book tests, PBL, Retention

## Abstract

The influence of problem-based learning (PBL) and open-book tests on long-term knowledge retention is unclear and subject of discussion. Hypotheses were that PBL as well as open-book tests positively affect long-term knowledge retention. Four progress test results of fifth and sixth-year medical students (n = 1,648) of three medical schools were analyzed. Two schools had PBL driven curricula, and the third one had a traditional curriculum (TC). One of the PBL schools (PBLob) used a combination of open-book (assessing backup knowledge) and closed-book tests (assessing core knowledge); the other two schools (TC and PBLcb) only used closed-book tests. The items of the progress tests were divided into core and backup knowledge. *T* tests (with Bonferroni correction) were used to analyze differences between curricula. PBL students performed significantly better than TC students on *core* knowledge (average effect size (av ES) = 0.37–0.74) and PBL students tested with open-book tests scored somewhat higher than PBL students tested without such tests (av ES = 0.23–0.30). Concerning backup knowledge, no differences were found between the scores of the three curricula. Students of the two PBL curricula showed a substantially better long-term knowledge retention than TC students. PBLob students performed somewhat better on core knowledge than PBLcb students. These outcomes suggest that a problem-based instructional approach in particular can stimulate long-term knowledge retention. Distinguishing knowledge into core and backup knowledge and using open-book tests alongside closed-book tests could enhance long-term *core* knowledge retention.

## Introduction

In preparing students to function as medical professionals, they have to be trained to become active, independent learners and problem solvers who are able to use and manage the growing and rapidly changing body of knowledge (Dolmans and Schmidt 1996; Heijne-Penninga et al. 2008a). Problem-based learning (PBL) is an instructional approach that suits these learning goals (Barrows and Tamblyn 1980; Dolmans and Schmidt 1996). Students in PBL curricula solve and discuss problems in small groups under supervision of a tutor. They are assumed to be better able to learn and recall information than students in more traditional curricula (Schmidt 1993; Dolmans and Schmidt 1996). However, studies on the effectiveness of PBL did not yield consistent results and, therefore, the effectiveness of PBL is still unclear and subject of substantial debate (Colliver 2000; Norman and Schmidt 2000; Kirschner et al. 2006; Schmidt et al. 2007; Hartling et al. 2010; Schmidt et al. 2012).

Assessment programmes also influence students’ learning approaches and, consequently, their knowledge acquisition (Cohen-Schotanus 1999; Van der Vleuten and Schuwirth 2005). A way to assess how students process the expanding body of knowledge is the use of open-book tests (Heijne-Penninga et al. 2008a). During these tests the students are allowed to consult their references, if they feel a need to do so. The availability of reference sources influences the ways students prepare for open-book tests and handle knowledge (Theophilides and Koutselini 2000; Broyles et al. 2005; Heijne-Penninga et al. 2008b). The influence of open-book tests on long-term knowledge retention is not yet known.

In this study, we investigated the effect of a PBL curriculum, combined with open-book tests, on students’ long-term knowledge retention.

### Memory and information processing

Knowledge required to be retained and recalled in the long term must be stored in memory. This memory storage process involves three successive stages: sensory memory, working memory and long-term memory (Atkinson and Shiffrin 1968). The *sensory memory* receives input from the senses—presumably before it is recognized—and information is retained temporarily in the sensory registers. This memory enables one to look at something and remember what it looked like with just a second of observation. The *working memory* provides a means of using knowledge and could therefore be considered as the most active part of the memory system. This memory allows one to recall information for a period of several seconds to a minute without rehearsal. The capacity of the working memory is limited: it can hold no more than five to nine elements of information, and even fewer elements—possibly no more than two or three—can be processed simultaneously (Miller 1956; Kirschner 2002; Paas et al. 2002). In both sensory and working memory, information is only available for a certain period of time. The *long*-*term memory* is the repository for more permanent knowledge and skills—information that is not currently being used, but is necessary for understanding (Bower 1975). This memory can store much more information than the previously discussed stages of memory, for potentially unlimited duration.

From the cognitive load theory we learn that knowledge is stored in the long-term memory as schemata, cognitive structures that make up an individual’s knowledge base (Sweller 1988). When a schema is retrieved from the long-term memory by the working memory, it can be handled as a single element, irrespective of its complexity. Consequently, schemata are not only used to organize knowledge, but also reduce the working memory load. Schemata are developed over a lifetime of learning and practice. They can be constructed in several ways: by combining elements during the process of problem-solving, by incorporating elements in schemata that already exist, and by obtaining schematized information from others (Van Merrienboer and Sweller 2010). These mental activities can be influenced by the teaching methods and assessment formats.

### PBL and open-book tests

Three important aspects of a PBL curriculum are context, constructivism and collaboration (Dolmans and Schmidt 1996). Solving problems in context stimulates elaboration on newly acquired knowledge, which in turn can promote the construction of cognitive schemata in the long-term memory (Schmidt 1993; Dolmans and Schmidt 1996). When prior knowledge is activated, mental models can be rebuilt or expanded. Problem-solving in small groups can also stimulate schemata construction, in particular when the presented problems are tailored to the students’ knowledge levels (Van Merrienboer and Sweller 2010). All three aspects of PBL can stimulate schemata construction and knowledge storage in the long-term memory. Therefore, we expect PBL to influence long-term knowledge retention positively.

In addition to teaching methods, assessment formats can also influence students’ learning approaches and, consequently, promote knowledge storage in their long-term memory. The long-term effect of employing open-book tests has not yet been investigated. When students prepare for an open-book test they need to focus on the body of knowledge as a whole. It is particularly important where information can be found and what the relationships are between various pieces of information. The ability to find and apply knowledge indicates a broader view of the learning content and more focus on interrelationships, which would result in a better way of structuring information and constructing comprehensive mental schemata (Driscoll 2005). Therefore, we expect open-book test preparation to influence long-term knowledge retention positively.

### Hypotheses

Earlier studies comparing the knowledge levels of PBL students and students in traditional curricula were based on scores on a single (national) knowledge test, for which the students had prepared extensively (Colliver 2000; Dochy et al. 2003; Distlehorst et al. 2005; Hoffman et al. 2006; Schuwirth et al. 2010; Hartling et al. 2010). This preparation aspect implies that the researchers assessed short-term rather than long-term knowledge retention. The outcome measures in our study, however, were progress test scores. The progress tests aim to assess knowledge that *graduated* medical doctors should master. These tests are not linked to an educational unit or to specific learning material and, therefore, students do not specifically prepare for progress tests (Van der Vleuten et al. 2004). Because of the time span between studying the knowledge and taking the test—usually, knowledge was studied in the first three study years—test results of fifth and sixth-years students can be considered to indicate long-term knowledge retention.

As explained before, both PBL and open-book tests are expected to influence long-term knowledge retention positively. We formulated the following hypotheses:

1. At the end of the curriculum, PBL students will perform better on progress tests than students in a traditional curriculum (TC).
2. At the end of the curriculum, students in a PBL curriculum with open-book tests will perform better on progress tests about backup knowledge than students in a PBL or TC curriculum without open-book tests.

## Method

### Participants and context

Progress test scores of fifth and sixth-year students of three medical schools (number of students = 1,648) were included in this study. The first medical school has a problem-based learning curriculum with both closed and open-book tests (PBLob, number of students = 600), the second medical school has a problem-based learning curriculum with only closed-book tests (PBLcb, number of students = 540) and the third medical school has a more traditional curriculum with only closed book tests (TC, number of students = 508).

All three medical schools offer a 6-year programme. The knowledge-based Bachelor’s programmes comprise pre-clinical training, whereas the clinically oriented Master’s programmes involve several clerkships. The three medical schools share the same learning objectives, which are set out in the National Blueprint for the Medical Curriculum (Van Herwaarden et al. 2009). The student populations of the three schools are highly similar as a result of the entrance procedure. Due to national policy, admission to Dutch medical schools is determined by a lottery system, weighted by grade point average, and students have limited influence on which university they will attend (Schuwirth et al. 2010).

The PBLob curriculum includes many small-group meetings, in which the students discuss and solve patient problems. Relatively few lectures are given and the basic and clinical sciences are taught in an integrated way. Since open-book tests are used next to closed-book tests, the total body of knowledge is divided into core and backup knowledge. Core knowledge is defined as the knowledge that any graduated medical doctor should dispose of without having to consult reference sources, and is assessed using closed-book questions. Backup knowledge is defined as the knowledge that a graduated medical doctor should be able to understand and apply properly with the use of reference sources, if necessary, and is assessed using open-book questions. Each knowledge test has a closed and an open-book component. After the closed-book answers are handed in, the students are allowed to open their reference sources to complete the open-book part of the test.

The PBLcb curriculum is comparable to the above-mentioned PBLob curriculum. However, PBLcb students have more freedom to pursue their own learning goals. Knowledge is not divided into core and backup knowledge, but is solely assessed using closed-book questions.

In the more traditional curriculum (TC) lectures are given on a regular basis and small group learning occurs less frequently than in the PBL curricula. The basic and clinical sciences are partly integrated and students are not confronted with patient problems on a regular basis. Knowledge is not divided into core and backup knowledge, but is solely assessed using closed-book questions.

### Progress test

In this study, students’ knowledge retention was measured with progress tests. Four times per year all medical students (years 1 through 6) of the participating three Dutch universities simultaneously sit the same progress test. Each progress test is composed of a different sample of 200 multiple choice items, with their content designed to comply with the Dutch National Blueprint for the Medical Curriculum (Van Herwaarden et al. 2009; Schuwirth et al. 2010). An item that has been part of a progress test will be locked for 3 years in the item bank before it may be reused. The progress tests aim to assess the functional knowledge that recently graduated medical doctors should master, using questions of different order (recall, application, analysis, et cetera). Furthermore, the progress tests provide students with feedback on their progress and possible gaps in medical knowledge during the 6-year curriculum, and are curriculum-independent (Schuwirth et al. 2010). During the tests, students are not allowed to consult their references and therefore progress tests are closed-book tests.

A single progress test consists of 200 multiple-choice questions, constructed by teaching faculty of all participating medical schools. Each test is jointly produced in accordance with the Blueprint, which ensures stratification of the sample by discipline and disease or complaint categories. At each participating medical school, the questions constructed by the school’s faculty are reviewed by a committee. When a question is approved by the local review committee, it enters the central item bank and can be sampled for inclusion in a future progress test. Examination regulations ensure comparable test status across schools (Muijtjens et al. 2007, 2008). On aggregate level, the test results can be used to identify differences in student performance between and within undergraduate medical curricula of the different schools (Muijtjens et al. 2008b).

Almost all theoretical knowledge that medical students acquire in the three curricula is studied in the Bachelor’s programme. Because of the time span between studying the knowledge and taking the test, test results of fifth and sixth-year students were interpreted as long-term performance.

### Procedure

Scores of fifth and sixth-year students on the four progress tests in the academic year 2008–2009 were included in this study. Questions that were eliminated for statistical and content reasons were left out of the analysis. The curriculum coordinator (JBMK), who was responsible for the content and construction of the entire PBLob curriculum, indicated for each question whether it focused on core (previously studied for closed-book tests) or backup knowledge (previously studied for open-book tests), as defined in the PBLob curriculum. The coordinators, who were responsible for the subsequent blocks in this curriculum, were asked to indicate for each progress test question whether it covered the theory of their block and whether it concerned core or back-up knowledge.

### Analysis

The content of each progress test was divided into two subtests: core knowledge and backup knowledge. Core knowledge referred to the knowledge all students had prepared for closed-book tests during their Bachelor’s programme. Backup knowledge referred to the knowledge PBLob students had prepared for open-book tests and PBLcb and TC students had prepared for closed-book tests during their Bachelor’s programme. For all subtests, a percentage-correct-minus-penalty-for-guessing score (formula scoring) was calculated for each cohort (year group) of each medical school (Rowley and Traub 1977). We compared students’ scores on core and backup knowledge between the three medical schools. To avoid complications due to the interdependence of scores, the analyses were performed separately for each of the four progress tests. *T* tests were used to compare percent-correct scores of all subtests. Score differences between the three schools (3 comparisons) were analyzed for four test moments and 2 year groups (years 5 and 6), resulting in 24 comparisons. To correct for this high number of multiple comparisons we used Bonferroni-corrected alpha equal to 0.05/24. Accordingly, for a comparison to be interpreted as statistically significant *p* ≤ 0.0021 was required. In addition, effect sizes (Cohen’s d) were calculated to indicate the importance of a difference. For each year group the weighted average of the effect sizes over the four test moments was calculated. According to Cohen’s classification, effect sizes of 0.2, 0.5, and 0.8, were considered to indicate small, medium, and large effects, respectively (Cohen 1988).

## Results

The 4 subtests on backup knowledge consisted of 109, 120, 120 and 122 questions, respectively; the 4 subtests on core knowledge consisted of 83, 65, 67 and 62 questions, respectively. In total, 26 out of 774 questions (3.4 %) were excluded from our analysis, because these questions concerned knowledge that could not clearly be classified as core or backup knowledge. The percent-correct scores on the subtests are presented in Table 1. Students’ performance on the four tests moments was better on core than on backup knowledge (paired *t* test, *p* < 0.0001), for each of the 2 year groups at all three medical schools.

**Table 1:** Mean scores per cohort, per medical school for each progress test

	Test 1 September 08		Test 2 December 08		Test 3 February 09		Test 4 May 09	
	Backup^a^	Core^a^	Backup^a^	Core^a^	Backup^a^	Core^a^	Backup^a^	Core^a^
Year 5								
PBLob	35.03	58.37	39.51	56.21	39.13	55.12	43.06	57.79
PBLcb	34.25	52.15	39.80	51.65	40.94	53.06	44.17	58.62
TC	32.25	49.24	38.71	47.92	37.81	47.11	41.22	52.94
Year 6								
PBLob	39.63	62.73	45.37	61.01	44.66	60.32	45.56	60.25
PBLcb	40.59	60.33	46.11	57.54	45.33	57.13	46.15	60.92
TC	38.40	55.75	44.96	53.84	43.32	54.44	44.70	57.37

All score differences Backup versus Core statistically significant (two-sided paired *t* test; *p* < 0.0001

^a^Backup = the mean score on the subtest concerning backup knowledge, Core = the mean scores on the subtest concerning core knowledge

### PBL versus TC

PBLob and PBLcb students significantly outperformed TC students on core knowledge (Table 2). Differences between TC and PBLob students were medium to high for seven of the eight test moments (av ES = 0.58–0.74). Differences between TC and PBLcb were small to medium for all eight test moments (av ES = 0.37–0.43).

**Table 2:** Core knowledge: comparison of the mean scores of the three medical schools

	PBLob versus PBLcb			PBLob versus TC			PBLcb versus TC		
	Diff	ES	av ES	Diff	ES	av ES	Diff	ES	av ES
Year 5									
Test 1	6.22*	0.62	0.30	9.13*	0.86	0.74	2.91*	0.28	0.43
Test 2	4.56*	0.47		8.28*	0.85		3.72*	0.36	
Test 3	2.07	0.20		8.02*	0.76		5.95*	0.56	
Test 4	−0.83	−0.08		4.85*	0.45		5.68*	0.54	
Year 6									
Test 1	2.40	0.28	0.23	6.98*	0.69	0.58	4.58*	0.45	0.37
Test 2	3.47*	0.39		7.17*	0.75		3.70*	0.40	
Test 3	3.19*	0.36		5.88*	0.59		2.69*	0.28	
Test 4	−0.67	−0.07		2.89	0.28		3.50*	0.35	

av ES: weighted average of the effect sizes over Tests 1–4

* *p* ≤ 0.0021 (one sided *t* test)

TC students also scored lower than PBLob and PBLcb students on backup knowledge, however, the effect sizes were small (av ES = 0.10–0.24) (Table 3).

**Table 3:** Backup knowledge: comparison of the mean scores of the three medical schools

	PBLob versus PBLcb			PBLob versus TC			PBLcb versus TC		
	Diff	ES	av ES	Diff	ES	av ES	Diff	ES	av ES
Year 5									
Test 1	0.79	0.08	−0.07	2.79*	0.28	0.17	2.00	0.21	0.24
Test 2	−0.29	−0.03		0.80	0.08		1.09	0.12	
Test 3	−1.81	−0.21		1.32	0.15		3.13*	0.34	
Test 4	−1.12	−0.12		1.83	0.20		2.95*	0.32	
Year 6									
Test 1	−0.97	−0.10	−0.08	1.23	0.13	0.10	2.20	0.22	0.19
Test 2	−0.74	−0.08		0.41	0.04		1.14	0.12	
Test 3	−0.67	−0.09		1.34	0.15		2.01	0.24	
Test 4	−0.58	−0.07		0.86	0.10		1.44	0.17	

av ES: weighted average of the effect sizes over Tests 1–4

* *p* ≤ 0.0021 (one sided *t* test)

### Open-book versus closed-book tests

PBLob students’ scores on backup knowledge did not significantly differ from those of PBLcb and TC students (Table 3). Fifth and sixth-year TC students scored lowest on backup knowledge. Of the eight tests moments, the difference with PBLob students was once significant (ES = 0.28) and the difference with PBLcb was twice significant (ES = 0.34 and 0.32) (Table 3).

## Discussion

In this study, we examined medical students’ long-term performance on progress tests. The results showed that PBL students outperformed TC students on core knowledge of most progress tests. Students from the PBLob curriculum, who had been assessed during their Bachelor’s programme—the first 3 years of the 6-year curriculum,—with both open-book tests on backup knowledge and closed-book tests on core knowledge, did not outperform their peers of the PBLcb and the TC curriculum on backup knowledge. However, they did perform better on core knowledge than their peers of both other curricula.

The hypothesis, that PBL students will perform better than TC students in the long term, was confirmed by our results. It seems that discussing knowledge in small groups and in the context of patient problems during the Bachelor’s programme can result in better knowledge retention at the end of the Master’s programme. The results of our study differed from those of studies in which scores on national tests—one point measures—were used as outcome measures (Vernon and Blake 1993; Colliver 2000; Hartling et al. 2010). Those studies found no or even a negative effect of PBL on knowledge level. A possible explanation for finding this difference might be, that the students had thoroughly prepared themselves to pass the national test shortly before the test was taken, which indicates a focus on short-term knowledge retention. Possibly, PBL students have difficulties with short-term retrieval of information that has been stored in their long-term memory, because they need specific retrieval cues that correspond to the patient problems discussed during the small-group meetings. Retrieval cues activate elements in long-term memory so they can be retrieved in working memory. If people have been exposed to the wrong cues, they can have difficulties activating a memory (Anderson 1995). Students in a PBL curriculum are confronted with knowledge that is presented in a specific context and linked with a specific patient problem. These contexts and problems might differ from those the test items are dealing with. Since the TC students have concentrated more on isolated knowledge, they might be less dependent of specific cues and, therefore, be better able to retrieve that knowledge in the short term. However, this isolated knowledge might be harder to retrieve in the long term, because it has not been embedded in and connected with other information elements in long-term memory (Byrnes 1996). This may have hampered TC students’ long-term knowledge retention and could explain their lower scores at the end of the Master’s programme.

The hypothesis that PBLob students would perform better on backup knowledge in the long term, was not confirmed by our data. During their Bachelor’s programme, PBLob students had prepared this type of knowledge for open-book tests. Because open-book tests are focused on the total amount of knowledge, we expected PBLob students to develop a broader view of the learning content. This would provide a better way of structuring information which, in turn, would result in better long-term knowledge retention (Driscoll 2005). A possible reason for not meeting these expectations may be that the students did not gain a broader overview of the knowledge, due to a lack of open-book test preparation. A previous study showed that bachelor students prepared less thoroughly for open-book than for closed-book tests (Heijne-Penninga et al. 2008b). Less thorough preparation for open-book tests can hamper the development of more elaborate schemata, which might be the reason for not finding differences between students who had prepared the backup knowledge for open or for closed-book tests.

The results of our study show, that PBLob students scored somewhat higher on core knowledge than PBLcb and TC students did. Possibly, as a result of the distinction between core and backup knowledge in their Bachelor’s programme, PBLob students had paid more attention to core knowledge. These students had prepared the core knowledge for closed-book tests, however, it was also part of the open-book tests. Consequently, they had been more exposed to the core knowledge than their peers in the two other curricula. Another reason for finding somewhat higher scores of PBLob students on core knowledge could be, that PBLob students were able to focus more on acquiring core knowledge, because the body of knowledge they had to know by heart was smaller. Besides, they possibly considered the closed-book tests to be more important and were therefore more motivated to study the core knowledge. In the PBLcb and TC curricula no distinction was made between core and backup knowledge, so in general the students were equally motivated to study all knowledge. However, the differences between PBLob and PBLcb students’ scores on core knowledge were small.

In this study, we compared progress test scores of students participating in three different curricula. Inter-curricular comparisons face some potential confounding issues and sources of error (Schmidt 1990; Colliver 2000; Norman 2003). To prevent bias, we used the progress test which is curriculum independent as an outcome measure, because it focuses on final objectives that are shared by all participating schools (Van Herwaarden et al. 2009). All three medical schools in our study were involved in test item construction, as advised by Muijtjens et al. (2007). The design also incorporated several measurements (four progress tests). Under these conditions, test results can be used to compare student achievement across different curricula (Muijtjens et al. 2007). We included progress test scores of a large number of students in our study and their knowledge levels were highly comparable due to the national admission procedure (Schuwirth et al. 2010). This study adds to existing research on PBL by focusing on actual long-term knowledge retention, using a curriculum independent knowledge test for which students did not specifically prepare (Van der Vleuten et al. 2004). Further research is needed in other curricula and in different contexts to generalize the results of our study. Besides, future research could concentrate on differences between clinical knowledge and basic sciences.

A possible limitation of this study is that the content of core and backup knowledge was determined by one medical school. Teachers of the other two medical schools might have different opinions about the content of core knowledge. However, their students also performed much better on the core knowledge parts of the tests. Their lower scores on backup knowledge indicate that our distinction between core and backup knowledge might be relevant. Two-thirds of the progress test questions concern backup knowledge, which raises the question whether the aim of the progress test to assess mastery of the functional knowledge that every graduating physician should possess is met (Schuwirth et al. 2010). The questions arise whether the progress test actually assesses the most relevant and required knowledge, and whether progress tests should contain open-book questions.

We did not correct for attrition and study duration. These two variables were indicated by Schmidt et al. (2012) as variables that mask positive effects of PBL. Their study revealed that, when correcting for these variables, reanalysis of the original data showed medium-level effect sizes favouring a PBL curriculum, whereas analysis without correcting for these two variables showed no effect of PBL on student performance. Therefore, the positive effect of PBL on knowledge retention we found could even be larger when the data is corrected by attrition and study duration.

A limitation to our study design is, that the study lacks a traditional curriculum with open-book tests (TCob), while this could have completed the design. Because such curricula do not exist in the Netherlands, we were unable to include students participating in a TCob curriculum.

In conclusion, students’ long-term knowledge retention was positively influenced by a problem-based curriculum format. Using open-book tests next to closed-book tests improved students’ performance on core knowledge questions. Distinguishing knowledge into core and backup knowledge, and using a combination of closed and open-book tests, could positively influence students’ core knowledge retention in the long term. Future research should concentrate on differences between clinical knowledge and basic sciences.

## References

[CR1] Anderson JR (1995). Learning and memory: An integrated approach.

[CR2] Atkinson RC, Shiffrin RM, Spence KW, Spence JT (1968). Human memory: A proposed system and its control processes. The psychology of learning and motivation: Advances in research and theory.

[CR3] Barrows HS, Tamblyn RM (1980). Problem-based learning: An approach to medical education.

[CR4] Bower, G. H. (1975). Cognitive psychology: an introduction. In W. K. Estes (Ed.), *Handbook of learning and cognitive processes (Vol. 1). Introduction to concept and issues* (pp. 25–80). Hillsdale, NJ: Erlbaum.

[CR5] Broyles IL, Cyr PR, Korsen N (2005). Open book tests: Assessment of academic learning in clerkships. Medical Teacher.

[CR6] Byrnes JP, Byrnes JP (1996). Memory. Cognitive development and learning in instructional contexts.

[CR8] Cohen J (1988). Statistical power analysis for the behavioral sciences.

[CR9] Cohen-Schotanus J (1999). Student assessment and examination rules. Medical Teacher.

[CR10] Colliver JA (2000). Effectiveness of problem-based learning curricula: Research and theory. Academic Medicine.

[CR12] Distlehorst LH, Dawson E, Robbs RS, Barrows HS (2005). Problem-based learning outcomes: The glass half-full. Academic Medicine.

[CR13] Dochy F, Segers M, Bosche PV, Gijbels D (2003). Effects of PBL: A meta-analysis. Learning and Instruction.

[CR14] Dolmans D, Schmidt H (1996). The advantages of problem-based curricula. Postgraduate Medical Journal.

[CR15] Driscoll M (2005). Psychology of learning for instruction.

[CR16] Hartling L, Spooner C, Tjosvold L, Oswald A (2010). Problem-based learning in pre-clinical medical education: 22 years of outcome research. Medical Teacher.

[CR17] Heijne-Penninga M, Kuks JBM, Hofman WHA, Cohen-Schotanus J (2008). Influence of open and closed-book tests on medical students’ learning approaches. Medical Education.

[CR18] Heijne-Penninga M, Kuks JBM, Schönrock-Adema J, Snijders TAB, Cohen-Schotanus J (2008). Open-book tests to complement assessment-programmes: Analysis of open and closed-book tests. Advances in Health Sciences Education.

[CR19] Hoffman K, Hosokawa M, Blake R, Headrick L, Johnson G (2006). Problem-based learning outcomes: Ten years of experience at the University of Missouri-Columbia School of Medicine. Academic Medicine.

[CR21] Kirschner PA (2002). Cognitive load theory: Implications of cognitive load theory on the design of learning. Learning and Instruction.

[CR22] Kirschner PA, Sweller J, Clark RE (2006). Why minimal guidance during instruction does not work: An analysis of the failure of constructivist, discovery, problem-based, experimental, and inquiry-based teaching. Educational Psychologist.

[CR24] Miller GA (1956). The magical number seven, plus or minus two: Some limits on our capacity for processing information. Psychological Review.

[CR25] Muijtjens AMM, Schuwirth LWT, Cohen-Schotanus J, Thoben AJNM, van der Vleuten CPM (2008). Benchmarking by cross-institutional comparison of student achievement in a progress test. Medical Education.

[CR26] Muijtjens AMM, Schuwirth LWT, Cohen-Schotanus J, van der Vleuten CPM (2007). Origin bias of test items compromises the validity and fairness of curriculum comparisons. Medical Education.

[CR27] Muijtjens AMM, Schuwirth LWT, Cohen-Schotanus J, van der Vleuten CPM (2008). Differences in knowledge development exposed by multi-curricular progress data. Advances in Health Sciences Education.

[CR28] Norman G (2003). RCT = results confounded and trivial: The perils of grand educational experiments. Medical Education.

[CR29] Norman GR, Schmidt HG (2000). Effectiveness of problem-based learning curricula: Theory, practice and paper darts. Medical Education.

[CR30] Paas F, Renkl A, Sweller J (2002). Cognitive load theory and instructional design: Recent developments. Educational Psychologist.

[CR31] Rowley GL, Traub RE (1977). Formula scoring, number-right scoring, and test-taking strategy. Journal of Educational Measurement.

[CR32] Schmidt HG, Nooman ZM, Schmidt HG, Ezzat ES (1990). Innovative and conventional curricula compared: What can be said about their effects?. Innovation in medical education: An evaluation of its present status.

[CR33] Schmidt HG (1993). Foundation of problem-based learning: Some explanatory notes. Medical Education.

[CR35] Schmidt HG, Loyens SMM, van Gog T, Paas F (2007). Problem-based learning is compatible with human cognitive architecture: Commentary on Kirschner, Sweller and Clark (2006). Educational Psychologist.

[CR36] Schmidt HG, Muijtjens AMM, Van der Vleuten CPM, Norman GR (2012). Differential student attrition and differential exposure mask effect of problem-based learning in curriculum comparison studies. Academic Medicine.

[CR37] Schuwirth L, Bosman G, Henning RH, Rinkel R, Wenink ACG (2010). Collaboration on progress testing in medical schools in the Netherlands. Medical Teacher.

[CR38] Sweller J (1988). Cognitive load during problem solving: Effects on learning. Cognitive Science.

[CR39] Theophilides C, Koutselini M (2000). Study behavior in the closed-book and the open-book test: A comparative analysis. Educational Research and Evaluation.

[CR40] Van der Vleuten CPM, Schuwirth LWT (2005). Assessing professional competence: From methods to programs. Medical Education.

[CR41] Van der Vleuten CPM, Schuwirth LWT, Muijtjens AMM, Thoben AJNM, Cohen-Schotanus J, van Boven CPA (2004). Cross institutional collaboration in assessment: A case on progress testing. Medical Teacher.

[CR42] Van Herwaarden CLA, Laan RFJM, Leunissen RRM (2009). The 2009 framework for undergraduate medical education in the Netherlands.

[CR43] Van Merrienboer JJG, Sweller J (2010). Cognitive load theory in health professional education: Design principles and strategies. Medical Education.

[CR44] Vernon DTA, Blake RL (1993). Does problem-based learning work? A meta-analysis of evaluative research. Academic Medicine.

